# Effect of a health education program on teachers' practices regarding *Helicobacter pylori* prevention among basic school teachers in Koya district

**DOI:** 10.3389/fpubh.2026.1830639

**Published:** 2026-06-10

**Authors:** Rukhosh Ishaq Mikha, Kareem Fattah Aziz

**Affiliations:** 1 Community Health Nursing Department, College of Nursing, Hawler Medical University, Erbil, Iraq; 2 Nursing Department, Koya Technical Institute, Erbil Polytechnic University, Erbil, Iraq

**Keywords:** health education, *Helicobacter pylori*, Iraq, preventive practices, teachers

## Abstract

**Background and aim:**

*Helicobacter pylori* infection remains a major public health concern, particularly in developing countries where poor hygiene facilitates transmission. Teachers serve as community role models; however, their preventive behaviors toward *H. pylori* infection are often insufficiently addressed. This study aimed to assess the association between a health education program and teachers' preventive practices regarding *H. pylori* infection among basic school teachers in Koya District, Erbil City, Iraq.

**Method:**

This quasi-experimental study was conducted from September 1st, 2024, to June 1st, 2025, across 10 basic schools in Koya District using a multistage sampling technique (cluster-based school selection from five geographical zones followed by random teacher selection). Data were collected from 104 teachers (52 intervention, 52 control) using a structured questionnaire developed from published guidelines and validated through expert panel review (S-CVI = 0.91). Statistical analyses included chi-square, *t*-tests, multiple linear regression, binary logistic regression, and mixed-effects regression.

**Results:**

Significant improvements were observed in the intervention group, particularly in filtered water use (Δ = 0.46, *p* < 0.01), clean water tank maintenance (Δ = 0.23, *p* = 0.03), and regular health check-ups (Δ = 0.58, *p* < 0.01). The intervention was associated with higher post-test practices scores (Adjusted R^2^ = 0.39, p < 0.001). Intervention participants were nearly four times more likely to demonstrate significant improvement (adjusted OR = 3.89, *p* = 0.003).

**Conclusions:**

The health education program was associated with improved teachers' preventive practices toward *H. pylori* infection. These findings suggest that structured, school-based health education may enhance hygiene practices, though confirmation through randomized multi-site studies with objective outcome measures and longer follow-up is warranted.

## 1 Introduction

*Helicobacter pylori (H. pylori)* infection remains a critical public health concern, yet preventive behavioral practices among educators and community figures have historically been insufficiently addressed (1). Globally, more than 4 billion people are estimated to be infected with *H. pylori*, making it one of the most prevalent bacterial infections in the world, particularly in low- and middle-income nations (2). Inconsistent hygienic behaviors, limited knowledge, and the absence of regular screening contribute to wide-ranging prevalence estimates of 30% to 80% in various regions (1, 2). Although the infection is distributed worldwide, teachers in resource-limited settings, who often serve as informal health communicators, face unique challenges in maintaining and modeling healthy practices (3). In Iraq, local surveys indicate that up to 65% of adults harbor *H. pylori*, and improper food handling and poor hand hygiene are the most common transmission routes (4). Global health efforts to address gastric infection prevention were initiated by WHO in 2001; however, practical behavior-change components targeting community educators were not part of those frameworks (5, 6). Comprehensive behavior-focused health education initiatives were only introduced nearly 20 years later under the WHO 2021–2030 health promotion roadmap (5, 7). As a result, *H. pylori* prevention practices continue to be one of the most overlooked community-level health challenges (8, 9).

In contrast to general awareness efforts, the prevention of *H. pylori* infection requires consistent hygienic practices that are reinforced through education and environmental support (10). Research indicates that improper handwashing before meals and shared utensil use are associated with a 3–5-fold increase in *H. pylori* transmission risk (11). Teachers who engage in proper hygiene and demonstrate preventive behaviors can serve as key role models for students and communities (10, 12). Those lacking adequate training, however, may unknowingly perpetuate risky behaviors that sustain household or school-level transmission cycles (13). A cross-sectional study in the Kurdistan Region found that less than 45% of teachers consistently practiced safe water consumption or proper food storage (14). In rural and peri-urban areas, teachers are often not exposed to health education campaigns, resulting in misconceptions about infection sources and ineffective preventive action (15, 16). In addition, gender and age differences influence preventive behavior. These findings highlight the need to translate knowledge improvements into tangible daily practices that reduce infection risk (17).

Current prevention guidelines emphasize routine handwashing, safe food preparation, and avoidance of shared utensils to prevent *H. pylori* spread (18). However, observational studies show that even after awareness programs, fewer than 50% of participants sustain proper hygienic practices for more than 6 months without reinforcement (19). Interactive health education, demonstration sessions, and periodic monitoring have emerged as key strategies to improve behavioral outcomes (19, 20). Although such interventions have shown measurable success in controlled settings, implementation in community and school environments remains limited due to resource and supervision constraints (19, 20). In Koya District, teachers have reported that although they receive general health information during school meetings, practical training on infection prevention behaviors is rarely included (21). Despite these developments, sustained improvement in daily preventive practices remains a challenge across most educational institutions (19, 22).

Given the high prevalence of *H. pylori* infection and the importance of preventive behavior, it is essential to evaluate whether structured educational programs are associated with improved teachers' infection-control practices (23). Teachers' health practices not only affect their own wellbeing but also influence students' hygiene behaviors and community health norms (24, 25). Studies from similar educational contexts have demonstrated that targeted training is associated with enhanced daily hygiene practices (26, 27). Moreover, reinforcement and follow-up sessions have been shown to maintain positive behavior change over time (28, 29). While previous studies have evaluated H. pylori knowledge and attitudes among various populations, there is a notable absence of evidence regarding whether structured educational interventions can improve actual preventive practices among school teachers in resource-limited settings such as the Kurdistan Region of Iraq. To date, no intervention study has specifically targeted teachers' daily hygiene and prevention behaviors related to H. pylori in this context. The present study, therefore, aims to assess the association between a health education program and teachers' practices regarding *Helicobacter pylori* prevention among basic school teachers in Koya District.

## 2 Research question

What is the association between a health education program and teachers' practices regarding *Helicobacter pylori* prevention among basic school teachers in Koya District?

## 3 Methods

### 3.1 Study design, setting, period, and sampling

This study employed a quasi-experimental design to evaluate the association between a health education program and *Helicobacter pylori* prevention practices among basic school teachers in the Koya District, Erbil City, Iraq. It was conducted from September 1st, 2024, to June 1st, 2025. A multistage sampling technique was employed. In Stage 1, 10basic schools were selected from five geographical zones (central, northern, southern, western, and eastern Koya District) using purposive cluster sampling to ensure geographic representation across the district. Two schools were selected from each zone. In Stage 2, all eligible teachers within these 10 schools were invited, yielding a pool of 200 eligible participants, from which 104 teachers were randomly selected using the lottery method. This school-level purposive selection, while practical and geographically representative, may limit generalizability to schools not included in the sampling frame. This is acknowledged as a limitation.

### 3.2 Sample size

The sample size was calculated using G^*^Power software (version 3.1.9.4) based on an independent-samples t-test (two-tailed), with an anticipated medium effect size (Cohen's d = 0.50), an alpha error probability of 0.05, and a statistical power of 0.80. The minimum required sample size was 102 participants (51 per group). To account for potential dropout, 104 teachers were enrolled (52 per group). The 104 teachers were divided equally into intervention (*n* = 52) and control (*n* = 52) groups. Allocation concealment was maintained using sealed, opaque, sequentially numbered envelopes prepared by a research assistant not involved in data collection. To minimize contamination, intervention and control participants were assigned to separate schools within different geographical zones, and teachers in each school were not informed about the parallel group at other schools. However, complete prevention of contamination cannot be guaranteed in a district-based design.

### 3.3 Inclusion and exclusion criteria

#### 3.3.1 Inclusion criteria

Basic school teachers currently employed in the selected schools who voluntarily agreed to participate and were available throughout the intervention and data collection period.

#### 3.3.2 Exclusion criteria

Teachers who had previously participated in a similar health education program; those who were part of the pilot study; those who were ill or absent during the intervention; those unwilling to participate; and those holding a degree in science (because their formal coursework in microbiology, infectious disease, and hygiene principles would create a baseline knowledge ceiling that could confound the assessment of the educational intervention's effect on non-science educators, who are the primary target for capacity building). This exclusion limits generalizability to all basic school teachers and is acknowledged as a limitation.

### 3.4 Study tools and data collection

The questionnaire was divided into three main sections: (1) sociodemographic and health-related information, (2) health-related behaviors, and (3) preventive practices related to H. pylori (14 items, rated Never/Sometimes/Always).

The 14-item practices questionnaire was developed based on published H. pylori prevention guidelines, peer-reviewed literature on hygiene behavior assessment, and existing validated instruments for food and water safety. Content validity was established through a panel of five experts (two community health nursing faculty, one gastroenterologist, one epidemiologist, one health education specialist). I-CVI ≥ 0.80 for all items; S-CVI/Ave = 0.91. Face validity was established through pilot testing with 20 teachers who confirmed that all items were clear, relevant, and culturally appropriate. Formal construct validity (e.g., factor analysis) was not conducted due to the small sample size; this is a limitation. The questionnaire was translated into Kurdish and Arabic using forward–backward translation. Data were collected through face-to-face interviews (15–20 min). The study was open-label; neither participants nor the researcher were blinded to group allocation.

#### 3.4.1 Description of the health education intervention

The health education program consisted of four structured educational sessions delivered over four consecutive weeks (one session per week), each lasting 45–60 minutes. Session 1 covered the fundamentals of H. pylori infection, including transmission routes, symptoms, and complications. Session 2 focused on safe water and food handling practices, with demonstrations of proper water filtration, food storage, and utensil hygiene. Session 3 addressed personal hygiene, including handwashing technique with return demonstration, nail hygiene, and avoiding shared personal items. Session 4 covered health-seeking behavior, including the importance of regular health check-ups, early symptom recognition, and treatment adherence. Pedagogical methods included interactive lectures with PowerPoint slides, group discussions, practical demonstrations with return demonstrations, video materials showing proper hygiene techniques, and a question-and-answer period at the end of each session. Each participant in the intervention group received a printed educational booklet (in Kurdish) summarizing all session content, serving as a take-home reinforcement resource. The control group received no educational intervention during the study period and continued routine school activities.

### 3.5 Pilot study

The questionnaire was tested with 20 teachers between April 1st and May 1st, 2024. Cronbach's alpha was 0.90 for the practices section, indicating very good reliability. Expert feedback confirmed suitability. Pilot data were excluded from the final analysis.

### 3.6 Measures

Sociodemographic and health-related characteristics, behavioral measures (smoking, alcohol, diet, physical activity), and preventive practices (14 items, Never/Sometimes/Always) were assessed as described in Section 3.4.

### 3.7 Ethical approval and informed consent

Ethical approval was obtained from the Ethics Committee of the College of Nursing, Hawler Medical University (Approval No. 248, dated 13 June 2024). The study was conducted in accordance with the Declaration of Helsinki. Written informed consent was obtained from all participants. Participation was voluntary; anonymity and confidentiality were maintained.

### 3.8 Statistical analysis

Data were summarized Using frequency, percentage, mean, and standard deviation. Group comparisons used Chi-square tests and independent-sample *t*-tests. The analytical strategy comprised both prespecified and exploratory approaches. Prespecified (primary) analyses: (1) paired-sample *t*-tests for within-group pre–post comparisons; (2) independent-sample *t*-tests for between-group comparison of post-test scores; (3) multiple linear regression (Models 1–3) to assess the intervention effect while progressively adjusting for confounders. Exploratory (secondary) analyses: (4) binary logistic regression; (5) mixed-effects regression; (6) Clinical Decision Support Thresholds (Table 1, *post-hoc*, not externally validated). The primary inferential basis is the stepwise linear regression (Table 2); the remaining models serve as supportive sensitivity analyses. We acknowledge that running multiple models on a sample of 104 participants carries a risk of over-analysis, and readers should interpret the exploratory models (Tables 3, 4, 1) with appropriate caution. Model performance used Nagelkerke R^2^, Hosmer–Lemeshow, and AUC. No missing data; all 104 participants completed pre- and post-test. SPSS 27.0; *p* < 0.05; all VIF < 3.0.

**Table 1:** Clinical decision support thresholds for enhancing *H. pylori* prevention practices among basic school teachers.

Practice category	Criteria (based on post-intervention and regression results)	*n* (approx.)	Practice characteristics	Key indicators	Recommended educational action
Very high	Intervention group + high baseline awareness + large practice improvement (Δ ≥4 points; post-score ≥38)	~25	Excellent hygiene, consistent handwashing, minimal risky food/water behaviors	*t* ≥5.0; *Cohen's d* ≥0.8; R^2^ >0.45	Assign as peer mentors and resource teachers for *H. pylori* prevention programs
High	Intervention group + moderate knowledge gain + positive attitude (Δ = 2.5–3.9; post-score 35–37)	~30	Good preventive habits, occasional lapses in water safety or check-ups	*t* = 3–4.9; *d* ≈ 0.6–0.7	Provide quarterly refreshers emphasizing screening and early detection
Moderate	Control group + partial improvement without intervention (Δ = 1.5–2.4; post-score 33–34)	~25	Acceptable practice; variable hygiene consistency and dietary risks	*t* ≈ 2–3; *d* ≈ 0.4–0.5	Schedule semi-annual workshops on hygiene reinforcement and dietary risk awareness
Low	Control group or intervention with low baseline practice (Δ ≤ 1.4; post-score < 33)	~15	Limited awareness; irregular handwashing; infrequent health checks	*t* < 2; *d* < 0.3; baseline < 25th percentile	Implement focused small-group sessions and personalized follow-up
Very low	No awareness of *H. pylori* or persistent misconceptions despite program	~9	Poor preventive behavior; reliance on self-medication; lack of water hygiene	OR < 1.0; β < 0.10; *p* > 0.05	Intensive counseling with visual materials and repeated on-site demonstration training

Δ, (post-test – pre-test practice score); *t*, paired-sample t-statistic; d, Cohen's d effect size; OR, odds ratio; β, standardized coefficient. Thresholds derived from Tables – showing strongest predictors of post-intervention practice: intervention group (β = 0.37, *p* < 0.001), pre-test practice score (β = 0.36, *p* < 0.001), and fast-food consumption (β = −0.16, *p* = 0.04). Model 3 of the multiple regression explained 47.6 % of variance in post-intervention practice scores.

**Table 2:** Multiple linear regression analysis predicting post-intervention practice scores among basic school teachers (*N* = 104).

Predictor variable	B	SE	β	t	*p*-value	95% CI
**Model 1: unadjusted**				
Intervention group (ref: control)	2.94	0.52	0.44	5.65	< 0.001	[1.91, 3.97]
Constant	34.86	0.37	–	94.22	< 0.001	[34.13, 35.59]
**Model summary: R**^2^ = **0.194, adjusted R**^2^ = **0.186, F (1,102)** = **31.93**, ***p***<**0.001**						
**Model 2: partially adjusted**						
Intervention group (ref: control)	2.68	0.49	0.40	5.47	< 0.001	[1.71, 3.65]
Pre-test practice score	0.38	0.07	0.39	5.43	< 0.001	[0.24, 0.52]
Age (years)	−0.01	0.02	−0.04	−0.50	0.62	[−0.05, 0.03]
Gender (ref: male)	0.56	0.48	0.08	1.17	0.24	[−0.39, 1.51]
Educational qualification (ref: diploma)	0.89	0.47	0.13	1.89	0.06	[−0.04, 1.82]
Teaching experience (years)	0.02	0.02	0.07	0.87	0.39	[−0.02, 0.06]
Constant	18.45	2.41	–	7.65	< 0.001	[13.67, 23.23]
**Model summary: R**^2^ = **0.389, adjusted R**^2^ = **0.352, F (6,97)** = **10.29**, ***p***<**0.001**						
**Model 3: fully adjusted**						
Intervention group (ref: control)	2.45	0.51	0.37	4.80	< 0.001	[1.44, 3.46]
Pre-test practice score	0.35	0.07	0.36	5.00	< 0.001	[0.21, 0.49]
Age (years)	−0.02	0.02	−0.07	−0.93	0.35	[−0.06, 0.02]
Gender (ref: male)	0.48	0.47	0.07	1.02	0.31	[−0.45, 1.41]
Educational qualification (ref: diploma)	0.82	0.46	0.12	1.78	0.08	[−0.09, 1.73]
Teaching experience (years)	0.03	0.02	0.10	1.25	0.21	[−0.01, 0.07]
Urban residence (ref: rural/suburban)	1.12	0.62	0.13	1.81	0.07	[−0.11, 2.35]
Family history of *H. pylori* (ref: no/unsure)	0.94	0.68	0.10	1.38	0.17	[−0.41, 2.29]
Previous awareness of *H. pylori* (ref: no)	1.48	0.78	0.14	1.90	0.06	[−0.07, 3.03]
Fast food consumption—Yes (ref: no)	−1.23	0.58	−0.16	−2.12	0.04	[−2.38, −0.08]
Fast food consumption—Sometimes (ref: no)	−0.54	0.52	−0.08	−1.04	0.30	[−1.57, 0.49]
Physical activity (ref: no)	0.67	0.45	0.10	1.49	0.14	[−0.22, 1.56]
Smoking status (ref: no)	−0.89	0.72	−0.09	−1.24	0.22	[−2.32, 0.54]
Coffee consumption (ref: no)	0.34	0.46	0.05	0.74	0.46	[−0.57, 1.25]
General health status (per category increase)	0.28	0.31	0.06	0.90	0.37	[−0.33, 0.89]
Constant	16.32	2.98	–	5.48	< 0.001	[10.41, 22.23]
**Model summary: R**^2^ = **0.476, adjusted R**^2^ = **0.392, F (15,88)** = **5.66**, ***p***<**0.001**						

Data are presented as mean (standard deviation). Statistical analysis performed using paired-sample *t*-test. *p* < 0.05 considered statistically significant. Cohen's d effect size interpretation: d = 0.2 (small), d = 0.5 (medium), d = 0.8 (large).

**Table 3:** Logistic regression analysis of factors associated with significant practice improvement following health education intervention (*N* = 104).

Predictor variable	Crude OR (95 % CI)	*p*-value	Adjusted OR (95% CI)	*p*-value
Intervention characteristics	
Intervention group (ref: control)	4.56 (2.02, 10.29)	< 0.001	3.89 (1.58, 9.57)	0.003
Sociodemographic factors	
Age ≥40 years (ref: < 40)	0.92 (0.43, 1.97)	0.83	0.81 (0.33, 1.98)	0.64
Female gender (ref: male)	1.52 (0.71, 3.28)	0.28	1.38 (0.58, 3.31)	0.47
Bachelor degree (ref: diploma)	1.96 (0.91, 4.23)	0.09	1.74 (0.72, 4.18)	0.22
Urban residence (ref: rural/suburban)	2.18 (0.81, 5.88)	0.12	1.89 (0.63, 5.67)	0.26
Teaching experience ≥15 years (ref: < 15)	1.23 (0.58, 2.62)	0.59	1.08 (0.46, 2.53)	0.86
Married (ref: single/widowed/separated)	1.67 (0.54, 5.15)	0.37	1.42 (0.41, 4.92)	0.58
Baseline practice level	
Pre-test practice score (per unit)	0.88 (0.78, 0.99)	0.04	0.86 (0.75, 0.98)	0.03
Low baseline practice (< 25th percentile) (ref: ≥25th)	3.24 (1.38, 7.60)	0.007	2.87 (1.14, 7.22)	0.025
Health awareness factors	
Family history of *H. pylori* (ref: no/unsure)	2.89 (0.85, 9.85)	0.09	2.34 (0.62, 8.85)	0.21
Previous awareness of *H. pylori* (ref: no)	3.12 (0.91, 10.70)	0.07	2.45 (0.64, 9.37)	0.19
History of *H. pylori* infection (ref: no/unsure)	1.78 (0.48, 6.58)	0.39	1.52 (0.37, 6.23)	0.56
Health status	
Good/very good health (ref: fair/poor)	1.43 (0.60, 3.42)	0.42	1.26 (0.48, 3.31)	0.64
Chronic disease (ref: no)	0.74 (0.27, 2.04)	0.56	0.68 (0.22, 2.10)	0.50
Current medication use (ref: no)	0.82 (0.32, 2.10)	0.68	0.78 (0.27, 2.23)	0.64
Behavioral risk factors	
Fast food consumption—yes (ref: no)	0.48 (0.17, 1.38)	0.17	0.42 (0.13, 1.35)	0.14
Fast food consumption—sometimes (ref: NO)	0.76 (0.30, 1.92)	0.56	0.71 (0.26, 1.96)	0.51
Physical activity (ref: no)	1.92 (0.89, 4.13)	0.10	1.68 (0.72, 3.92)	0.23
Smoking (ref: no)	0.58 (0.16, 2.09)	0.40	0.52 (0.13, 2.08)	0.36
Coffee consumption (ref: no)	1.34 (0.63, 2.87)	0.45	1.18 (0.51, 2.75)	0.70
Tea consumption (ref: no)	1.56 (0.50, 4.86)	0.45	1.32 (0.38, 4.59)	0.66
NSAID use (ref: no/sometimes)	0.89 (0.33, 2.40)	0.82	0.84 (0.28, 2.52)	0.76
Model fit statistics	
Nagelkerke R^2^			0.508	
−2 log likelihood			102.47	
Model χ^2^ (df = 19)			43.89 (*p* = 0.001)	
Hosmer–Lemeshow χ^2^ (8)			7.23 (*p* = 0.51)	
AUC (95% CI)			0.84 (0.76, 0.91)	
Sensitivity/specificity/accuracy			79.6%/76.3%/78.8%	

R^2^ = Multiple correlation coefficient squared; Adj. R^2^ = Adjusted R^2^; ΔR^2^ = Change in R^2^; df1 = degrees of freedom for variables added; df2 = residual degrees of freedom; F-change = significance test of ΔR^2^; *p* < 0.05; ^*^p < 0.01; ^**^p < 0.001. Standardized β coefficients (Beta weights) from the final step (Step 4) show the relative importance of predictors. All VIF values in the final models were below 3.0, indicating no multicollinearity concerns.

**Table 4:** Mixed-effects regression analysis of practice score changes over time by intervention status (*N* = 104).

Effect/parameter	B	SE	*t*/z	*p*-value	95% CI	Additional statistics
Fixed effects	
Intercept	33.24	1.89	17.59	< 0.001	[29.51, 36.97]	
Time (post-test vs. pre-test)	1.78	0.34	5.24	< 0.001	[1.11, 2.45]	
Group (intervention vs. control)	0.58	0.52	1.12	0.27	[−0.45, 1.61]	
Time × group interaction	2.46	0.48	5.13	< 0.001	[1.51, 3.41]	
Covariates	
Age (years)	−0.02	0.02	−1.02	0.31	[−0.06, 0.02]	
Gender (female vs. male)	0.52	0.46	1.13	0.26	[−0.39, 1.43]	
Educational qualification (bachelor vs. diploma)	0.86	0.45	1.91	0.06	[−0.03, 1.75]	
Urban residence (vs. rural/suburban)	1.08	0.60	1.80	0.07	[−0.11, 2.27]	
Teaching experience (years)	0.03	0.02	1.38	0.17	[−0.01, 0.07]	
Family history of *H. pylori* (yes vs. no/unsure)	0.89	0.66	1.35	0.18	[−0.41, 2.19]	
Previous awareness of *H. pylori* (yes vs. no)	1.42	0.76	1.87	0.06	[−0.08, 2.92]	
Fast-food consumption—yes (vs. no)	−1.18	0.56	−2.11	0.04	[−2.29, −0.07]	
Fast-food consumption—sometimes (vs. No)	−0.52	0.50	−1.04	0.30	[−1.51, 0.47]	
Physical activity (yes vs. no)	0.64	0.44	1.45	0.15	[−0.23, 1.51]	
Random effects	
Participant (intercept)	8.45	—	—	—	—	SD = 2.91; ICC = 0.38
Residual	13.82	—	—	—	—	SD = 3.72
Model fit statistics	
−2 log likelihood = 892.34 AIC = 932.34 BIC = 985.67						Marginal R^2^ = 0.42 Conditional R^2^ = 0.65
Simple slopes/estimated marginal means	
Control—pre-test	32.68 (0.65)				[31.40, 33.96]	
Control—post-test	34.46 (0.65)				[33.18, 35.74]	Change = +1.78^*^ (0.48) [0.83, 2.73]
Intervention—pre-test	33.26 (0.63)				[32.02, 34.50]	
Intervention—post-test	37.50 (0.63)				[36.26, 38.74]	Change = +4.24^***^ (0.47) [3.31, 5.17]
Difference in change (intervention—control)	+2.46^***^ (0.48)			< 0.001	[1.51, 3.41]	

B, unstandardized coefficient; SE, standard error; CI, confidence interval; ICC, intraclass correlation coefficient; AIC, Akaike Information Criterion; BI, Bayesian Information Criterion. *p* < 0.05, *p* < 0.01,^*^p < 0.001. A significant Time × Group interaction indicates that the intervention group exhibited a substantially greater improvement in practice scores compared with the control group. Marginal R^2^ represents variance explained by fixed effects; Conditional R^2^ reflects variance explained by both fixed and random effects. A random-intercept model accounted for repeated measures within participants.

## 4 Results

### 4.1 Sociodemographic and health-related characteristics

A total of 104 participants were included, equally divided between intervention and control groups. The mean age was 41.2 ± 8.6 years. Females slightly predominated (55.8% vs. 63.5%). Most were married (94.2% vs. 78.8%) and reported adequate income. Significant differences were noted in residence (*p* < 0.01) and family history of H. pylori (19.2% vs. 3.8%, *p* = 0.03) see Table 5.

**Table 5:** Sociodemographic and health-related characteristics of participants by study group (*N* = 104).

Variable	Category	Intervention group F (%)	Control group F (%)	*p*-value
Age (years)	< 30	–	7 (13.5)	–
	30–39	23 (44.2)	20 (38.5)	0.37
	40–49	17 (32.7)	18 (34.6)	
	≥50	12 (23.1)	7 (13.5)	
Gender	Male	23 (44.2)	19 (36.5)	0.09
	Female	29 (55.8)	33 (63.5)	
Marital status	Single	2 (3.8)	7 (13.5)	0.37
	Married	49 (94.2)	41 (78.8)	
	Widowed	1 (1.9)	2 (3.8)	
	Separated	–	2 (3.8)	
Monthly income	Not enough	–	4 (7.7)	–
	Enough for daily needs	45 (86.5)	43 (82.7)	0.30
	Exceeds daily needs	7 (13.5)	5 (9.6)	
Residence	Urban	45 (86.5)	45 (86.5)	< 0.01
	Suburban	6 (11.5)	1 (1.9)	
	Rural	1 (1.9)	6 (11.5)	
Qualification	Diploma institute	33 (63.5)	23 (44.2)	0.12
	Bachelor	19 (36.5)	29 (55.8)	
Teaching experience (years)	1–5	1 (1.9)	5 (9.6)	0.41
	6–10	8 (15.4)	14 (26.9)	
	11–15	14 (26.9)	9 (17.3)	
	16–25	19 (36.5)	19 (36.5)	
	≥26	10 (19.2)	5 (9.6)	
School region	Central	12 (23.1)	10 (19.2)	0.84
	Northern	10 (19.2)	12 (23.1)	
	Eastern	10 (19.2)	10 (19.2)	
	Western	10 (19.2)	10 (19.2)	
	Southern	10 (19.2)	10 (19.2)	
Heard about H. pylori	Yes	48 (92.3)	45 (86.5)	0.29
	No	4 (7.7)	7 (13.5)	
History of H. pylori infection (last 6 months)	Yes	2 (3.8)	6 (11.5)	0.08
	No	42 (80.8)	40 (76.9)	
	Not sure	8 (15.4)	6 (11.5)	
Family history of H. pylori	Yes	2 (3.8)	10 (19.2)	0.03
	No	48 (92.3)	37 (71.2)	
	Not sure	2 (3.8)	5 (9.6)	
General health status	Very good	4 (7.7)	4 (7.7)	0.60
	Good	34 (65.4)	30 (57.7)	
	Fair	9 (17.3)	18 (34.6)	
	Poor	5 (9.6)	–	
Chronic disease	Yes	10 (19.2)	9 (17.3)	0.79
	No	42 (80.8)	43 (82.7)	
Use of medication	Yes	9 (17.3)	15 (28.8)	0.16
	No	43 (82.7)	37 (71.2)	

Data are presented as frequency (percentage). *p*-values were calculated using the Chi-square test. Values with *p* < 0.05 were considered statistically significant. Missing or blank categories indicate unavailable comparison data.

### 4.2 Teachers' behavioral characteristics

Most participants did not smoke (84.6% vs. 90.4%) or drink alcohol (94.2% vs. 98.1%). Tea consumption was high (>85% in both groups). A significant difference was observed in fast-food consumption (*p* = 0.04), where 32.7% of the intervention group ate fast food regularly vs. 13.5% in controls see Table 6.

**Table 6:** Characteristics of teachers' behavior regarding the intervention and control groups (*N* = 104).

Behavior	Category	Intervention group F (%)	Control group F (%)	*p*-value
Do you smoke cigarettes?	Yes	8 (15.4)	5 (9.6)	0.32
	No	44 (84.6)	47 (90.4)	
If yes, how many cigarettes do you smoke per day?	5 cigarettes	1 (12.5)	–	–
	10 cigarettes	2 (25.0)	2 (40.0)	
	1 packet	2 (25.0)	2 (40.0)	
	More than one packet	3 (37.5)	1 (20.0)	
Do you smoke (hookah or shisha)?	Yes	–	1 (1.9)	0.31
	No	52 (100.0)	51 (98.1)	
Do you drink alcohol?	Yes	3 (5.8)	1 (1.9)	0.29
	No	49 (94.2)	51 (98.1)	
If yes, frequency of drinking	Weekly	3 (100.0)	–	–
	Monthly	–	1 (100.0)	
Do you drink coffee?	Yes	30 (57.7)	27 (51.9)	0.56
	No	22 (42.3)	25 (48.1)	
If yes, how many cups per day	One cup	24 (80.0)	21 (77.8)	–
	Two cups	4 (13.3)	5 (18.5)	
	Three cups	1 (3.3)	1 (3.7)	
	More than 3 cups	1 (3.3)	–	
Do you drink tea?	Yes	48 (92.3)	45 (86.5)	0.36
	No	4 (7.7)	7 (13.5)	
If yes, how many cups per day	One cup	7 (14.6)	7 (15.6)	–
	Two cups	7 (14.6)	15 (33.3)	
	Three cups	18 (37.5)	14 (31.1)	
	More than 3 cups	16 (33.3)	9 (20.0)	
Do you often consume fizzy drinks?	Yes	3 (5.8)	4 (7.7)	0.71
	Sometimes	16 (30.8)	30 (57.7)	
	No	33 (63.5)	18 (34.6)	
Do you take non-steroidal anti-inflammatory drugs (e.g., aspirin, ibuprofen)?	Yes	11 (21.2)	6 (11.5)	0.18
	Sometimes	–	4 (7.7)	
	No	41 (78.8)	42 (80.8)	
Do you often consume spicy foods?	Yes	20 (38.5)	13 (25.0)	0.21
	Sometimes	16 (30.8)	28 (53.8)	
	No	16 (30.8)	11 (21.2)	
How is your food overall in terms of saltiness?	Salted	1 (1.9)	1 (1.9)	0.92
	Normal	49 (94.2)	50 (96.2)	
	Unsalted	2 (3.8)	1 (1.9)	
Do you do any physical activity?	Yes	24 (46.2)	24 (46.2)	1.00
	No	28 (53.8)	28 (53.8)	
If yes, frequency of activity	Daily	10 (41.7)	4 (16.7)	–
	Few times a week	14 (58.3)	20 (83.3)	
Do you eat fast food?	Yes	17 (32.7)	7 (13.5)	0.04
	Sometimes	27 (51.9)	34 (65.4)	
	No	8 (15.4)	11 (21.2)	

Data are presented as frequency (percentage). *p*-values were obtained using the Chi-square test. Values of *p* < 0.05 were considered statistically significant. Dashes (–) denote unavailable data or categories not reported.

### 4.3 Teachers' practices regarding h. pylori prevention

Significant overall improvements **were observed** in preventive practices in the intervention group. The most substantial improvements occurred in using filtered water (Δ = 0.46, *t* = 2.92, *p* < 0.01, d = 0.81), using tap water only when safe (Δ = 0.65, *t* = 4.81, *p* < 0.01, d = 0.69), cleaning water tanks (Δ = 0.23, *t* = 2.16, *p* =0.03, d = 0.54), avoiding sharing utensils (Δ = 0.33, *t* = 2.84, *p* < 0.01, d = 0.59), avoiding infection sources (Δ = 0.27, *t* = 3.06, *p* < 0.01, d = 0.45), and regular health check-ups (Δ = 0.58, *t* = 4.99, *p* < 0.01, d = 0.59). Handwashing practices showed minimal change due to already high baseline levels (Table 7).

**Table 7:** Assessment of basic school teachers' practices regarding prevention of *H. pylori* infection in intervention and control groups (*N* = 104).

Item	Pre-test M (SD) intervention	Post-test M (SD) intervention	Pre-test M (SD) control	Post-test M (SD) control	Mean difference (int.)	*t* (int.)	*p*-value (int.)	Cohen's d (int.)
Have you ever tested for *H. pylori* infection?	1.53 (0.57)	1.96 (0.55)	1.55 (0.62)	1.73 (0.61)	0.42	3.80	< 0.01	0.57
Do you always wash your hands after using the toilet?	2.98 (0.13)	3.00 (0.00)	2.88 (0.40)	2.98 (0.13)	0.02	1.00	0.32	0.10
Do you wash your hands before eating?	2.90 (0.29)	2.98 (0.13)	2.83 (0.42)	2.90 (0.32)	0.08	1.69	0.09	0.23
Do you take precautions to avoid potential sources of *H. pylori* infection?	2.59 (0.53)	2.86 (0.34)	2.44 (0.63)	2.67 (0.51)	0.27	3.06	< 0.01	0.45
Do you frequently eat fast food, which may increase the risk of *H. pylori* infection?	3.96 (0.48)	4.50 (0.57)	4.06 (0.50)	4.31 (0.57)	0.54	5.16	< 0.01	0.53
Do you ensure clean water is your main source of drinking water?	2.00 (0.76)	2.32 (0.64)	2.20 (0.75)	2.38 (0.65)	0.33	2.35	0.02	0.71
Do you use filtered water for drinking?	1.80 (0.88)	2.26 (0.71)	1.98 (0.85)	2.27 (0.73)	0.46	2.92	< 0.01	0.81
Do you use tap water for drinking?	3.80 (0.68)	4.46 (0.69)	3.95 (0.74)	4.20 (0.72)	0.65	4.81	< 0.01	0.69
Do you regularly clean and maintain your home water tank?	2.23 (0.54)	2.46 (0.54)	2.16 (0.55)	2.35 (0.55)	0.23	2.16	0.03	0.54
Do you avoid sharing food utensils to prevent infection?	2.36 (0.62)	2.69 (0.54)	2.28 (0.69)	2.59 (0.54)	0.33	2.84	< 0.01	0.59
Do you undergo regular health check-ups to detect and prevent *H. pylori* infection?	1.57 (0.57)	2.15 (0.60)	1.63 (0.62)	1.94 (0.66)	0.58	4.99	< 0.01	0.59
Do you ensure proper disposal of sewage and garbage at home?	2.59 (0.60)	2.88 (0.37)	2.58 (0.64)	2.80 (0.46)	0.29	2.92	< 0.01	0.50
Do you take medications for stomach pain without consulting a doctor?	4.38 (0.69)	4.51 (0.70)	4.46 (0.69)	4.45 (0.70)	0.14	0.99	0.33	0.70
Do you ensure that food is properly cooked and safely stored?	1.98 (0.72)	2.50 (0.57)	2.03 (0.76)	2.42 (0.63)	0.52	4.03	< 0.01	0.66

Data are presented as mean (standard deviation). Statistical analysis performed using paired-sample *t*-test. *p* < 0.05 considered statistically significant. Cohen's d effect size interpretation: d = 0.2 (small), d = 0.5 (medium), d = 0.8 (large).

### 4.4 Multiple linear regression

In Model 1, intervention group membership was associated with increased post-test scores by 2.94 points (*p* < 0.001, R^2^ = 0.194). Model 2 (adjusted for demographics) showed B = 2.68, *p* < 0.001, R^2^ = 0.389. In the fully adjusted Model 3, B = 2.45, *p* < 0.001, R^2^ = 0.476. Fast-food consumption was negatively associated (B = −1.23, *p* =0.04) see Table 2.

### 4.5 Logistic regression

Intervention group membership was associated with nearly fourfold higher odds of significant practice improvement [adjusted OR = 3.89, 95% CI (1.58, 9.57), *p* = 0.003]. Lower baseline practice was also predictive (adjusted OR = 2.87, *p* = 0.025). Model: Nagelkerke R^2^ = 0.508, AUC = 0.84, classification accuracy = 78.8% (Table 3).

### 4.6 Mixed-effects regression

The Time × Group interaction was highly significant (B = 2.46, *p* < 0.001), confirming greater practice gains in the intervention group (+4.24, *p* < 0.001) vs. controls (+1.78, *p* < 0.05). Fast-food consumption was negatively associated (B = −1.18, *p* = 0.04). Model: marginal R^2^ = 0.42, conditional R^2^ = 0.65 see Table 4.

### 4.7 Clinical decision support thresholds

The results revealed five performance categories among teachers' preventive practices after the intervention. About 25 teachers (Very High) demonstrated excellent hygiene and consistent behaviors, showing the largest practice gains (Δ ≥4, *t* ≥ 5.0, d ≥0.8, R^2^ >0.45) and are ideal as peer mentors. Thirty teachers (High) showed notable improvement (Δ = 2.5–3.9) with good habits but minor lapses, suggesting quarterly refreshers. The Moderate group (~25) achieved partial progress (Δ = 1.5–2.4) and would benefit from semi-annual workshops. Fifteen teachers (Low) showed limited improvement (Δ ≤ 1.4) and need small-group training, while nine (Very Low) showed no improvement or persistent misconceptions, requiring intensive counseling and practical demonstrations see Table 1.

## 5 Discussion

The present study assessed the association between a health education program and teachers' preventive practices regarding *Helicobacter pylori* among basic school teachers in Koya District. Overall, the results revealed that the educational intervention was associated with significantly improved preventive practices, with participants in the intervention group demonstrating substantially higher post-test scores compared to controls, particularly in safe drinking water use, food utensil hygiene, and regular health check-ups.

*Helicobacter pylori* infection continues to pose a substantial global health burden, with transmission occurring primarily through contaminated water, food, and poor hygiene practices (30). In developing regions, including the Kurdistan Region of Iraq, inadequate sanitation infrastructure and limited awareness of preventive behaviors contribute to persistently high infection rates (31). Teachers occupy a unique position in communities, serving not only as educators but also as behavioral role models whose practices influence students and families (32). Despite their pivotal role, there is insufficient evidence regarding the association between targeted interventions and teachers' preventive practices against *H. pylori* in this region.

The demographic profile of participants was consistent with the typical composition of the basic education teaching workforce: a mean age of approximately 41 years, predominantly married females, with adequate income and relatively high baseline awareness of H. pylori (33, 34). The significant differences between groups in residence and family history of H. pylori infection are noteworthy and are addressed below in the context of confounding.

The substantial improvement in teachers' preventive practices following the intervention represents a critical finding. The significant increases in post-test scores, with particularly notable gains in safe drinking water use and food utensil hygiene, suggest that knowledge enhancement may translate into behavioral change when appropriately facilitated. These findings are consistent with previous studies reporting significant practice improvements following targeted health education (35). The moderate-to-large effect sizes (d = 0.45–0.81) suggest that the intervention addressed practical barriers to behavior change.

The regression analyses provided valuable insights. The finding that intervention group membership independently predicted practice scores, explaining nearly half the variance in the fully adjusted model, suggests a robust association beyond baseline characteristics. The negative association between fast-food consumption and practice improvement (36) highlights the importance of addressing broader lifestyle factors.

The logistic regression results suggesting that intervention group membership increased odds of improvement nearly fourfold underscore the intervention's substantial association with positive outcomes. The mixed-effects model confirmed that practice improvements were greater in the intervention group, with a significant Time × Group interaction.

An important finding requiring discussion is the significant improvement observed in the control group (+1.78 points, *p* < 0.05) despite not receiving the structured educational intervention. Several factors may explain this finding: (1) Cross-contamination: Although intervention and control groups were assigned to separate schools in different geographical zones, teachers within the same district may have shared information through social networks, professional meetings, or informal contact, leading to partial diffusion of educational content. (2) Hawthorne effect: Being enrolled in a study, completing questionnaires, and being observed by a researcher may have heightened awareness and self-monitoring of hygiene behaviors in both groups, independent of the intervention content. (3) Temporal and seasonal effects: The study period (September to June) coincides with the academic year, during which routine school health activities or public health campaigns may have influenced teachers' practices independently. (4) Testing effect: Repeated exposure to the practice questionnaire itself may have prompted reflection and behavior modification. These factors collectively highlight the inherent difficulty of fully isolating intervention-specific effects from broader environmental influences in quasi-experimental community-based designs.

Although regression models adjusted for baseline differences in residence, family history of H. pylori, and fast-food consumption, residual confounding from unmeasured variables (e.g., access to clean water infrastructure, household socioeconomic factors, exposure to health information outside the study) cannot be excluded. The significant baseline imbalance in family history of H. pylori infection (19.2% in controls vs. 3.8% in intervention, *p* = 0.03) is particularly noteworthy, as personal or familial experience with the infection may independently influence motivation and receptivity to health education. While statistical adjustment mitigates measured confounding, it cannot fully substitute for balanced randomization, and the observed associations should therefore be interpreted with appropriate caution.

Several important limitations must be acknowledged. First, the multistage sampling with purposive school selection at the cluster level and the exclusion of science-degree teachers limit generalizability. Second, the quasi-experimental design cannot establish causality with the same certainty as RCTs. Third, the study was open-label with no blinding of participants, assessors, or analysts. Fourth, all outcome measures relied on self-report, susceptible to social desirability bias. No objective validation measures were used, which is a major limitation. Fifth, the study captures only immediate post-intervention effects. The absence of long-term follow-up (e.g., 3–6 months) prevents assessment of whether observed improvements are sustained over time. Behavior-change literature consistently demonstrates that gains from educational interventions may diminish without periodic reinforcement. This is a major limitation and a priority for future research. Sixth, contamination between groups cannot be fully excluded. Seventh, formal construct validity was not assessed. Eighth, running multiple statistical models on a sample of 104 participants may constitute over-analysis; the primary regression (Table 4) should serve as the main inferential basis. Future research should incorporate RCT designs, blinded assessment, objective behavioral verification (e.g., handwashing observation, water quality testing, H. pylori stool antigen tests), longer follow-up, multi-site recruitment, and full psychometric evaluation.

## 6 Conclusion

The health education program was associated with improved teachers' preventive practices toward *H. pylori* infection. Teachers who participated showed gains in safe water consumption, food hygiene, and health check-up behaviors. However, these findings should be interpreted with caution given the quasi-experimental design, self-reported outcomes, absence of blinding, no objective verification, and no long-term follow-up. The results suggest that structured school-based health education may be associated with improved hygiene practices, but these findings require confirmation through randomized controlled trials with objective outcome measures, blinded assessment, and sustained follow-up before broader policy recommendations can be made (37–46).

## Data Availability

The original contributions presented in the study are included in the article/supplementary material, further inquiries can be directed to the corresponding author.

## References

[1] Elbehiry A Marzouk E Aldubaib M Abalkhail A Anagreyyah S Anajirih N . Helicobacter pylori infection: current status and future prospects. Antibiotics. (2023) 12:191. doi: 10.3390/antibiotics12020191 36830102 PMC9952126

[2] Li Y Choi H Leung K Jiang F Graham DY Leung WK. Global prevalence of Helicobacter pylori infection between 1980 and 2022. Lancet Gastroenterol Hepatol. (2023) 8:553–64. doi: 10.1016/S2468-1253(23)00070-5 37086739

[3] Borka Balas R Meli? LE Mărginean CO. Worldwide prevalence and risk factors of H. pylori in children. Children. (2022) 9:1359. doi: 10.3390/children9091359 PMC9498111 36138669

[4] Saeed AY Rashad BH Ali BN Sulaivany AH Ibrahim KS. H. pylori infection: prevalence, risk factors, and treatment efficacy in Zakho City, Iraq. Cureus. (2024) 16:e73873. doi: 10.7759/cureus.73873 39697942 PMC11652683

[5] Conti CB Agnesi S Scaravaglio M Masseria P Dinelli ME Oldani M . Early gastric cancer: update on prevention, diagnosis and treatment. Int J Environ Res Public Healt. (2023) 20:2149. doi: 10.3390/ijerph20032149 PMC9916026 36767516

[6] Mabe K Inoue K Kamada T Kato K Kato M Haruma K. Endoscopic screening for gastric cancer in Japan. Dig Endosc. (2022) 34:412–9. doi: 10.1111/den.14063 34143908

[7] Li Z Song X Zeng Y Gong C Luo YF Fang W . Regional Variation, Lifestyle Determinants, and Household Clustering of Helicobacter pylori in Coastal Southern China: A Multicenter, Population-Based Cross-Sectional Study.

[8] Hyun CS Oh SS Wu L Moy RH Shin JI. Rethinking gastric cancer prevention through an immigrant health lens. Nat Rev Gastroenterol Hepatol. (2025) 22:600–1. doi: 10.1038/s41575-025-01098-0 40691298

[9] Schuppler M. On the role of food in the transmission of *Helicobacter pylori* infection: a narrative review. Foods. (2025) 14:4325. doi: 10.3390/foods14244325 41465032 PMC12733334

[10] Zhou M Zeng Y Xi Y Luo S Qi J Zhao G . School-based Hygiene Intervention to Prevent HelicObacter Pylori infection among childrEn (SHIP HOPE): protocol for a cluster-randomised controlled trial. BMJ Open. (2022) 12:e064207. doi: 10.1136/bmjopen-2022-064207 PMC9772681 36600426

[11] Liu M Liu S Lu Z Chen H Xu Y Gong X . Machine learning-based prediction of H. pylori infection study. Med Sci Monit. (2024) 30:e943666–1. doi: 10.12659/MSM.943666 PMC11168235 38850016

[12] Abdelwahab DAA Abdelaty HIM Shaban MEA Ahmed DAAS. Knowledge and health beliefs regarding H pylori prevention among nursing students Egypt. J Nurs Health Sci. (2025) 6:55–75. doi: 10.21608/ejnhs.2025.456098

[13] Argueta EA Moss SF. How we approach difficult to eradicate H. pylori. Gastroenterology. (2022) 162:32–7. doi: 10.1053/j.gastro.2021.10.048 34743914

[14] Rostam RA Rashid HI Hassan SO Aziz KT Rashid HI Hassan SO . Prevalence of H. pylori among children in Sulaimani City Kufa. J Nurs Sci. (2024) 14:120–30. doi: 10.36321/kjns.vi202401.15571

[15] Maqbul MS Alshehri WAA Beig STM Brik RKb Alshehri AH Alamri MS . Prevalence of knowledge about H pylori among urban population of KSA. Gastroenterol Endosc. (2024) 2:196–204. doi: 10.1016/j.gande.2024.08.001

[16] Wang L Li ZK Lai JX Si YT Chen J Chua EG . Risk factors associated with H pylori in the urban population of China. Int J Infect Dis. (2025) 154:107890. doi: 10.1016/j.ijid.2025.107890 40096882

[17] Maude RR Jongdeepaisal A Skuntaniyom S Muntajit T Blacksell SD Khuenpetch W . Improving KAP to prevent COVID-19 transmission in Thailand *BMC Public Health*. (2021) 21:749. doi: 10.1186/s12889-021-10768-y PMC8053080 33865342

[18] Benjamen M. An Investigation into the Transmission of Helicobacter pylori through Food Prepared and Consumed under Hygienic and Unhygienic Conditions: A Pioneering Study Using Biopsy Samples. (2025).

[19] Yang Z Xiong W Yang R Qian H He Z Chen M . A day-to-day management model improves patient compliance for H pylori treatment. Gut Pathog. (2023) 15:38. doi: 10.1186/s13099-023-00556-x 37518066 PMC10388557

[20] Hafiz TA D'Sa JL Zamzam S Visbal Dionaldo ML Aldawood E Madkhali N . Effectiveness of an educational intervention on H. pylori for university students. J Multidiscip Healthc. 2023:1979–88. doi: 10.2147/JMDH.S419630 PMC10361274 37484821

[21] Rashid AA Othman SM. Assessment of KAP of health staff toward infection control in Erbil. Bahrain. Med Bull. (2023) 45.

[22] Ansari S Yamaoka YH. pylori: laboratory diagnosis and antimicrobial resistance. Clin Microbiol Rev. (2022) 35:e00258–21. doi: 10.1128/cmr.00258-21 35404105 PMC9491184

[23] Wang YX Zou JY Hu LF Liu Q Huang RL . Chinese public awareness of H. pylori screening. BMJ Open. (2022) 12:e057929. doi: 10.1136/bmjopen-2021-057929 PMC8796245 35078854

[24] El-maghawry A Metwaly SM Elpasiony NMA. H. pylori health literacy sessions on university students' knowledge. Egypt J Health Care. (2022) 13. doi: 10.21608/ejhc.2022.281021

[25] Gadekar U. Hygiene practices and community well-being in a rural setting. IJEMR. (2023) 13:99–104. doi: 10.31033/ijemr.13.4.13

[26] Gozdzielewska L Kilpatrick C Reilly J Stewart S Butcher J Kalule A . Effectiveness of hand hygiene interventions for community transmission prevention. BMC Public Health. (2022) 22:1283. doi: 10.1186/s12889-022-13667-y 35780111 PMC9250256

[27] Mushota O Mathur A Pathak A. Effect of school-based WASH intervention on students' knowledge. BMC Public Health. (2021) 21:2258. doi: 10.1186/s12889-021-12279-2 34895193 PMC8666030

[28] Kuponiyi A Akomolafe OO. A Digital Health Framework for Expanding Access to Preventive Services.

[29] Smith JE Arkin E Rosen B. Comprehensive Nutrition Therapy for Co-Occurring GI & Eating Disorders. 2025.

[30] Yuan C Adeloye D Luk TT Huang L He Y Xu Y . Global prevalence of H pylori in children. Lancet Child Adolesc Health. (2022) 6:185–94. doi: 10.1016/S2352-4642(21)00400-4 35085494

[31] Naqid IA Al-Brefkani A Hussein NR. Prevalence and risk factors for H pylori in Duhok Province. Arch Razi Inst. (2024) 79:272. doi: 10.32592/ARI.2024.79.2.272 39463724 PMC11512183

[32] Gao S Tang Z-C Miao H Li J Tang Z-B Liu J-H . Awareness and attitudes regarding H pylori among university students. Life Res. (2022) 5:1–8. doi: 10.53388/2022-0116

[33] Archampong TN. Helicobacter pylori Diversity and Gastro-Duodenal Disease in Western Africa (Doctoral dissertation). University of Leicester, Leicester (2021).

[34] Zandian H Zahirian Moghadam T Pourfarzi F Malekzadeh R Rezaei S Ghorbani S. Gastric troubles in Iran: social and economic factors in H pylori. Health Promot Perspect. (2023) 13:120. doi: 10.34172/hpp.2023.15 37600545 PMC10439454

[35] Zha J Li YY Qu JY Yang XX Han ZX Zuo X. Effects of enhanced education for H pylori patients: meta-analysis. Helicobacter. (2022) 27:e12880. doi: 10.1111/hel.12880 35150600

[36] Fosu B. Fast food consumption pattern of bankers in Kasoa, Ghana. U of Education Winneba. (2021).

[37] Simmons T. Risk Factors of H. pylori for Hispanics in Southern Arizona U of Arizona. (2024).

[38] Merryweather JA. Geographic pattern and socioecological factors of H. pylori in the US Walden U. (2023).

[39] Sun M Liu E Yang L Cao H Han M. Scoping review of worldwide guidelines for H pylori. Syst Rev. (2025) 14:107. doi: 10.1186/s13643-025-02816-0 40346683 PMC12063324

[40] Taber KS. The use of Cronbach's alpha when developing and reporting research instruments. Res Sci Educ. (2018) 48:1273–96. doi: 10.1007/s11165-016-9602-2

[41] Al Omari SM Khalifeh AH Moman R Sawan HM. KAP related to H. pylori and gastric disease in Jordan. Infect Drug Resist. (2025) 18:1503–14. doi: 10.2147/IDR.S508330 40123709 PMC11930260

[42] Chan EYY Tong KHY Dubois C Mc Donnell K Kim JH Hung KKC . Narrative review of primary preventive interventions against water-borne diseases. Int *J Environ Res Public Health*. (2021) 18:12268. doi: 10.3390/ijerph182312268 PMC8656607 34885995

[43] Ebrahimi Z Shateri Z Nouri M Sikaroudi MK Masoodi M Shidfar F . Ultra-processed food intake and risk of H pylori infection. Food Sci Nutr. (2024) 12:5019–26. doi: 10.1002/fsn3.4152 39055221 PMC11266909

[44] Shah SC Iyer PG Moss SF AGA. clinical practice update on refractory H. pylori Gastroenterology. (2021) 160:1831–41. doi: 10.1053/j.gastro.2020.11.059 33524402 PMC8281326

[45] Habbash F Alalwan TA Perna S Ahmed N Sharif O . Association between dietary habits and H pylori among Bahraini adults. Nutrients. (2022) 14:4215. doi: 10.3390/nu14194215 36235867 PMC9572631

[46] Socias-Pappaterra P Ebrahim Ibrahim YS Ho Sang V Moquete Grullón B Romano R Contreras C . Predicted probability of gastric intestinal metaplasia. ACG. (2025) 120:S266–267. doi: 10.14309/01.ajg.0001132400.63602.0f

